# Experimental study and finite element analysis of heavy-duty escalator truss under full load conditions

**DOI:** 10.1038/s41598-024-55175-6

**Published:** 2024-02-28

**Authors:** Ning Li, Zhongxi Cao, Wei Bao, Suixian Lin, Tao Zou, Mingming Yan

**Affiliations:** 1 School of Mechanical and Electrical Engineering, Guangzhou University, Guangzhou, 510000 People’s Republic of China; 2 Enterprise Development and Innovation Center, Guangzhou Guangri Elevator Industry Co., Ltd., Guangzhou, 510000 People’s Republic of China; 3 Escalator Technology Department, Guangzhou Guangri Elevator Industry Co., Ltd., Guangzhou, 510000 People’s Republic of China; 4 School of Civil Engineering, Guangzhou University, Guangzhou, 510000 People’s Republic of China

**Keywords:** Heavy-duty, Escalator truss, Deflection, Experimental study, Simulation analysis, Mechanical engineering, Mechanical properties

## Abstract

The performance of the heavy-duty escalator truss greatly affects the stability and service life of the whole escalator system, and the manufacturing cost of truss structure accounts for more than 1/5. Thus, how to design the truss structure reasonably is a pivotal issue drawing the attention of numerous engineers and researchers. In this work, the experimental research of heavy-duty escalators under full load conditions were performed in terms of the end restraints, the docking port clearances, and the deflection. Based on the experimental results, the three-dimensional simulation model of truss structure was created, and the influences of various factors such as the internal chamfer of truss member, the lower deviation of truss member, the dead weight of escalator, and the pretension force of each bolt at the docking port were analyzed and quantified. Finally, the finite element model which can almost completely characterize the actual structure was obtained with slight difference. The conclusions drawn in this work provide the basis for the efficient design, correct simulation, low cost production and rapid installation of the heavy-duty escalator truss.

## Introduction

An escalator is a type of fixed electric drive facility with circulating running steps, which incline up or down to transport passengers continuously^1^. Compared with the elevator, it has a greater theoretical transport capacity, which is widely used in places where people are concentrated such as commercial buildings, various public transport places, and so on. The birth of the escalator has provided great convenience for ordinary people in their daily travel. With the continuous development of urbanization construction, various large shopping malls and transportation facilities like railway stations, subways, and airports are constantly emerging, leading to the growing demand for escalators is also rising year by year, thus increasing the importance of escalators in people's daily life. Therefore, the safety of escalators has attracted more and more attention, and higher requirements for the escalator have been put forward.

Truss serves as the primary load-bearing framework of an escalator, which is used to connect the upper and lower adjacent floor planes, uphold the whole weight of the escalator structure and bear the load force for transporting passengers, etc. To ensure the safety and stability of heavy-duty escalator during operation, many escalator manufacturers often adopt oversized profile steels to produce the trusses based on traditional empirical design methods, which makes the strength and stiffness of the trusses too large, resulting in the cost of the truss accounting for more than 1/5. Especially, as the number of escalators increases, the problem of material usage in trusses is becoming more and more prominent. Thus, how to design the truss structure reasonably has become an urgent matter that needs to be addressed, on the premise of ensuring adequate strength and rigidity and concomitantly minimizing steel consumption.

However, the more in-depth research on truss structure was mainly focused on the building field. Zhou et al. investigated the response of an 8-story steel staggered-truss system, utilizing a scaled model at 1/8th size subjected to reversed low cyclic loading, and put forward some suggestions for the design of a staggered-truss structure^2^. Amélia et al. created a new flattening typology by incorporating stiffeners in the lateral edges of the diagonal flattened ends, and found that this stiffened flattening technique can simplify the structural design of the plane and multi‐planar trusses^3^. To replace traditional reinforcement methods for coupling beams used within shear wall system, Chairunnisa et al. modeled a simplified version of a coupling beam using steel truss configurations made from steel angle profiles and found that this kind of steel truss-coupling beam can show fairly well behavior under lateral load^4^. Wang et al. proposed an innovative approach with a cold-formed thin-walled steel tube truss shear wall reinforced with oriented strand board on both sides, aiming to meet the seismic capacity requirements for in low- and multi-rise buildings^5^. To investigate the behavior of pitched timber roof structures with long span stabilized by bracing trusses positioned in the plane of the top chord, Sejkot et al. performed three parametric studies and found that remarkable support forces may be generated in the compressed structural members of such long-span timber structures^6^. Liang et al. investigated the structure-borne noise characteristics and mitigation strategies associated with long-span steel truss cable-stayed bridges in urban rail transit systems, caused by train operations^7^. Güldür et al. focused on studying the bending property of cold-formed steel floor trusses constructed from lipped channel sections with concrete-filled compression chord member, and found the concrete infill within the compression chord member not only inhibited chord local/distortional buckling but also significantly enhanced the truss's performance by increasing its stiffness and load capacity^8^. Zhang et al. analyzed the effects of partition walls and strongbacks on the vertical vibration performance of full-scale 12m span tooth plates affixed to wood truss joist floors, and found they could efficiently diminish vertical displacement and acceleration at the floor's center under pedestrian load while augment the natural frequency^9^. Based on a steel truss arched bridge with long span, Liu et al. investigated the fatigue mechanism, cracking progression, and fatigue property assessment of an orthotropic steel bridge deck subjected to traffic loading^10^. Although these published research findings may play a reference role, they cannot directly guide the design of the escalator trusses.

In recent years, many customers who purchase or customize escalators put forward the requirements that the finite element analysis result files of the escalator truss must be provided. In addition, finite element method can help reduce the design costs, the consumption of materials, test time and expenses, etc. Therefore, the simulation analysis technique is extensively employed in the design process of escalator trusses. Entrusted by Suzhou Jiangnan Express Elevator Company, Zhang carried out structural analysis and optimization design of the escalator truss, and found that the transverse beam can reduce the stress and deformation of the truss with minor effect, and using the lower chord with a smaller section can reduce the waste of materials^11^. Based on the sensitivity analysis, the finite element analysis and optimization method, Zhang et al. redesign the elevator truss structure with the wall thickness of each component as the design variable and the stiffness and strength as the design constraints^12^. Using software REFEM, Liang analyzed the deflection and stress of the escalator trusses, and found that the results met the customer's specification requirements^13^. Wang calculated the deformation and stress of a truss under various load conditions, and found that the deformation of the truss met the requirements of the national standard, but the truss had local stress overrun^14^. Gao et al. studied the influence of transverse beams, and found that the transverse beams played a significant role in reducing the ship escalator truss deformation under the roll condition, and had little effect under the static condition with the same conclusions^15^ as in the literatures^11,16^. To carry out effective system design calculations for escalator truss structures, He et al. explored the mechanical properties of escalator truss in terms of the main influencing factors such as the use of profiles of different sizes in the upper or lower chord, the position distribution of skew beams, and different section heights of the truss^17^. To meet the needs for lightweight without compromising the safety of escalator truss structures, Xu investigated the mechanical characteristics of public transport escalator truss with different widths with one center support under full load conditions by simulation^18^. To improve efficiency, Tang optimized the section heights of the escalator truss to minimize the maximum deformation of the truss by using Altair HyperOpt Solver with the adaptive response surface method^19^. Yu and Dong made a comparative analysis of the statics simulation between the rectangular tube truss and the angle steel truss of the escalator, and found little difference in maximum deflection and strength between them^20^. Yang et al. carried out the mechanical simulation analysis on an escalator truss structure subjected to a range of loading scenarios, and found that the simulation results and the actual measured values were almost identical^21^. Furthermore, in order to maintain the escalator's superior safety performance during lightweight design initiatives in public transit systems, the structural rigidity and strength characteristics of the steps and truss for heavy-duty escalators under full load conditions were studied by simulation^22–24^.

Although the above researches have played a very good guiding role in the design of the escalator trusses, there are still many questions that need to be clarified and studied. Firstly, the escalator trusses were considered as a whole and fixed constraints were imposed on both ends of the trusses in all these simulation studies, which needs to be further studied because they do not conform to actual situations. Secondly, the influences of simplifying the truss members and the dead weight of the escalator were also neglected in these simulations, which may also have a relatively large impact. Thirdly, to save costs, material suppliers often provide profiles with the lower deviations to escalator truss manufacturers, but how much impact it will have is still unknown. Finally, it is well-known that the heavy-duty escalator truss is connected together by high strength bolts at the docking ports, and the pretension force of each bolt applied through the torque wrench is determined by experience, which is often inaccurate. How much preload is appropriate to apply is worth investigating. Yet up to now, relevant studies have rarely been reported. In this work, the above questions were explored by experimental study and finite element analysis.

The organization of this paper is as follows. Firstly, an experimental investigation on a heavy-duty escalator truss under full load conditions was conducted to determine the type of end restraints. However, due to the diversity of structural parameters, long production cycle, and high test cost of the escalator truss, an experimental study may not be suitable for conducting a comprehensive analysis. Therefore, finite element analysis was performed utilizing the ABAQUS software. Three-dimensional simulation models were created and validated based on the previous experimental results. Thirdly, the influences of various factors such as the internal chamfer, the lower deviation of the truss member, the dead weight of the escalator, and the pretension force of each bolt on the escalator truss were parametrically studied. Finally, drawing from the experimental and simulation results, some suggestions were put forward for efficient design, correct simulation, low cost production and rapid installation of the heavy-duty escalator truss.

## Experimental study

### Detailed information about the experimental object

**Figure 1:**
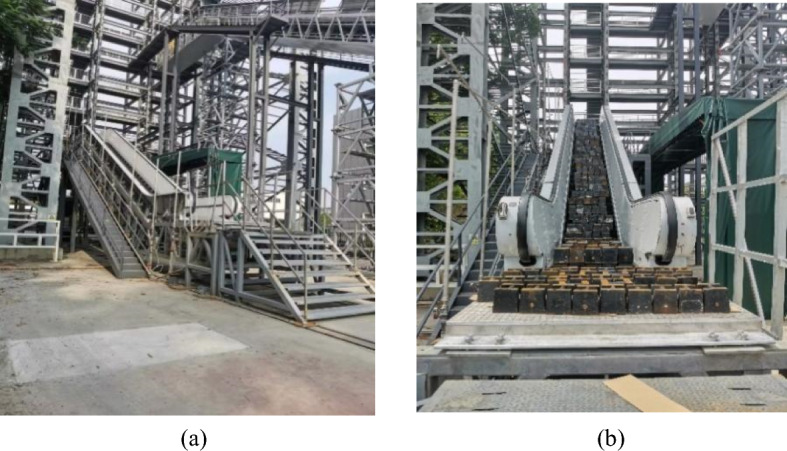
The experimental object: (**a**) before loading, and (**b**) after loading.

The experimental object in this work was a heavy-duty escalator without center support, which was supported on the steel frame platform by eight bolts on the end support beams as shown in Fig. 1. Its technical specifications are enumerated in Table 1. The lower extremity of the steel frame platform is fixed to the concrete ground by anchor bolts, and the upper end of the steel frame platform is welded to the fixed columns.

**Table 1:** Technical specifications of the heavy-duty escalator.

Parameter	Sign	Value
Rise of escalator	R_e_	5370 mm
Angle of inclination	A_i_	30°
Nominal width	N_w_	1000 mm
Truss width	T_w_	1690 mm
Horizontal span	H_s_	18,700 mm

Serving as the escalator's primary support, the truss is often designed as a segmental structure including a lower horizontal section, several straight sections, and an upper horizontal section, for the convenience of handling and transportation due to its high rise of the escalator. Under normal circumstances, the docking position of the truss is located in the middle inclined section with the advantages of strong versatility and operability, while also avoiding affecting the strength of important parts such as the turning points.

**Figure 2:**
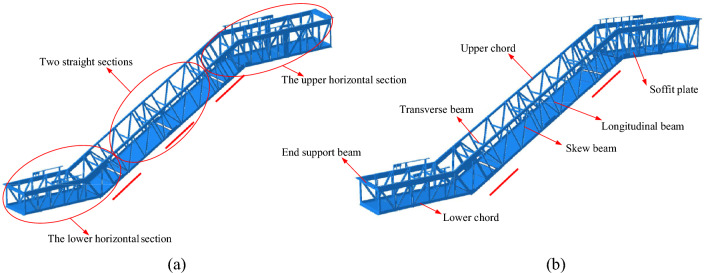
Truss structure: (**a**) with two straight sections, and (**b**) with one straight section.

Figure 2 shows two kinds of truss structure to analyze the influence of the intermediate docking port, one with two straight sections in Fig. 2a, and the other with one straight section in Fig. 2b. As can be seen, the truss is generally composed of the end support beams, the upper chords, the lower chords, the longitudinal beams, the skew beams, the transverse beams, the soffit plate, etc., which are connected into an integral structural frame by fully welding. The specific details of truss members are outlined in Table 2. Notably, all the members are constructed from steel grade Q235-B, which features a Young's modulus of 2.06 * 10^5^ MPa and a Poisson’s ratio of 0.3. The material density is 7850 kg/m^3^.

**Table 2:** The details of truss members.

Parameter values	Truss (a)/mm	Truss (b)/mm
Height of the lower horizontal section	1200	1200
Height of the straight section	1500	1650
Height of the upper horizontal section	1500	1500
The end support beams	∠200 * 200 * 24	∠200 * 200 * 24
The chords in the lower/upper horizontal section	∠125 * 80 * 8	∠125 * 80 * 8
The lower chords in the straight section	∠125 * 80 * 8	∠125 * 80 * 10
The upper chords in the straight section	∠125 * 80 * 10	∠125 * 80 * 10
The longitudinal beams in the lower/upper horizontal section	⊏100 * 48 * 5.3	⊏100 * 48 * 5.3
The longitudinal beams in the straight section	∠63 * 63 * 6	∠63 * 63 * 6
The skew beams in the lower/upper horizontal section	⊏80 * 43 * 5	⊏80 * 43 * 5
The skew beams in the straight section	∠63 * 63 * 6	∠63 * 63 * 6
The transverse beams	⊏63 × 40 × 4.8	⊏63 × 40 × 4.8
The soffit plate	5	5

### Experimental apparatus

**Figure 3:**
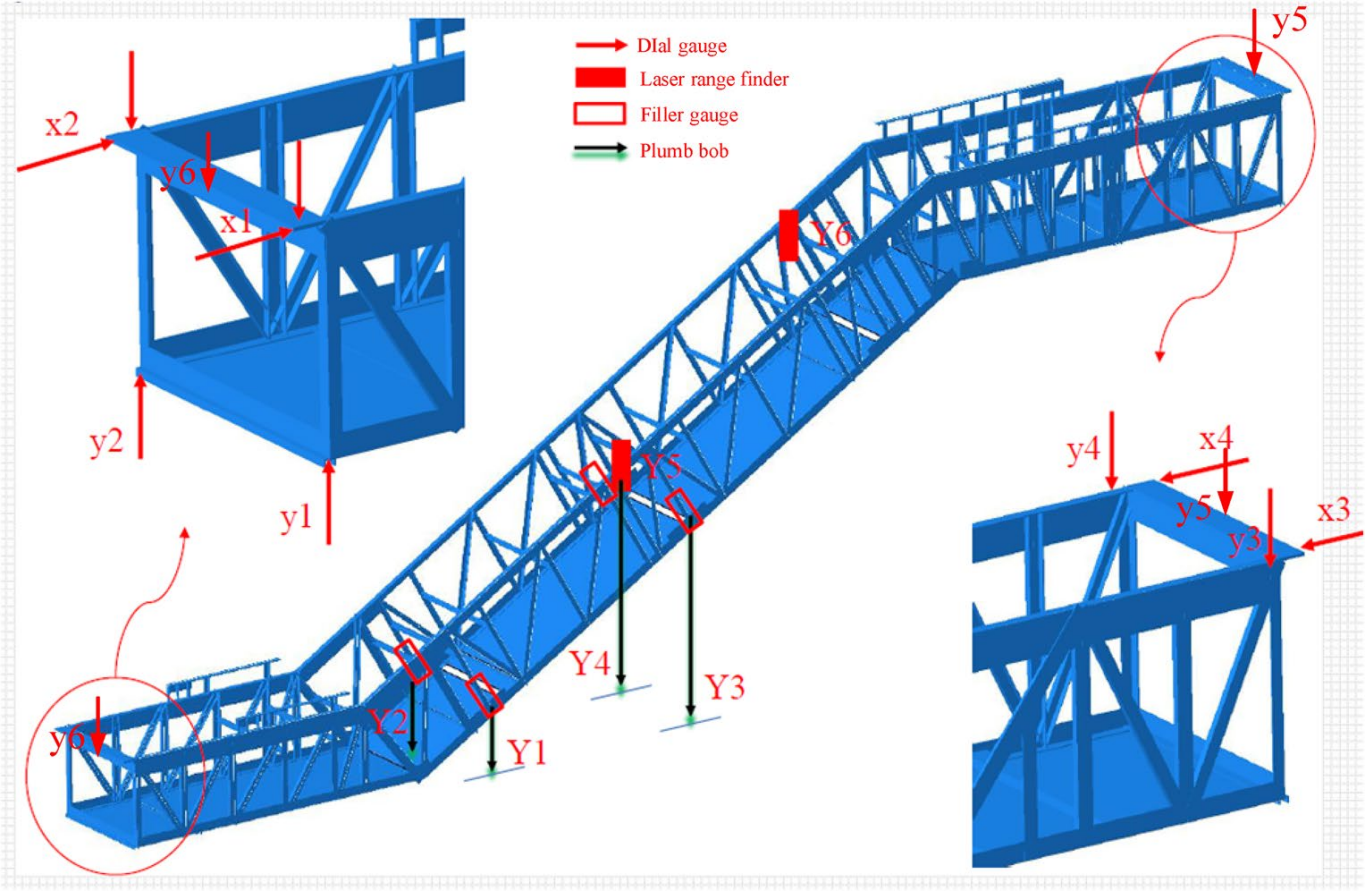
The test equipment and measuring points.

The main technical parameter of the truss is deflection. According to the standard requirements GB 16899^25^, the maximum deflection, whether calculated or measured, must not exceed 1/1500 of the horizontal span between the supporting points under the condition of applying a load of 5000 N/m^2^ on the horizontal projection area of the heavy-duty escalator. Therefore, the deflection requirements should be ensured first when designing the main structure of the truss. To verify whether the designed trusses meet the deflection requirements, the full load tests of the escalator were conducted, and the test equipment and measuring points were shown in Fig. 3, taking the truss structure with two straight sections for example. Ten dial gauges were fixed on the steel frame platform by magnetic suction to measure the horizontal slip and vertical displacement of positions x1–x4, and y1–y6, respectively. Typically, the maximum deflection may occur in the middle of the truss, therefore four plumb bobs were fastened to the longitudinal beams at positions Y1–Y4 through wire lines, to measure the vertical deformation at these positions. As comparison, two laser rangefinders were glued to the blank places near the upper and the lower chords at positions Y5 and Y6 to measure the vertical deformation of these positions. At the same time, the docking port clearances at the positions Y1–Y4 were measured by filler gauge.

The meanings of the above-measured values at each measuring point were summarized in Table 3. In this work, the relative measuring method was adopted, namely, the measured values at each measuring point were the relative values before and after loading.

**Table 3:** The meanings of the measured values at each measuring point.

Label	Position	Measured values	Test equipment
x1	The right side of the end support beam in the lower horizontal section	Horizontal slip	Dial gauge 1
x2	The left side of the end support beam in the lower horizontal section	Horizontal slip	Dial gauge 2
y1	The right-end longitudinal beam in the lower horizontal section	Vertical displacement	Dial gauge 3
y2	The left-end longitudinal beam in the lower horizontal section	Vertical displacement	Dial gauge 4
x3	The right side of the end support beam in the upper horizontal section	Horizontal slip	Dial gauge 5
x4	The left side of the end support beam in the upper horizontal section	Horizontal slip	Dial gauge 6
y3	The right-end longitudinal beam in the upper horizontal section	Vertical displacement	Dial gauge 7
y4	The left-end longitudinal beam in the upper horizontal section	Vertical displacement	Dial gauge 8
y5	The middle of the end support beam in the upper horizontal section	Vertical displacement	Dial gauge 9
y6	The middle of the end support beam in the lower horizontal section	Vertical displacement	Dial gauge 10
Y1	The right side of the lower docking port	Vertical displacement	Plumb bob 1
		Docking port clearance	Filler gauge
Y2	The left side of the lower docking port	Vertical displacement	Plumb bob 2
		Docking port clearance	Filler gauge
Y3	The right side of the intermediate docking port	Vertical displacement	Plumb bob 3
		Docking port clearance	Filler gauge
Y4	The left side of the intermediate docking port	Vertical displacement	Plumb bob 4
		Docking port clearance	Filler gauge
Y5	The left side of the intermediate docking port	Vertical displacement	Laser range finder 1
Y6	The left side of the upper docking port	Vertical displacement	Laser range finder 2

### Design of loading scheme

After being hoisted onto the steel frame platform, the experimental escalator should be adjusted to a normal operating state at first to facilitate the application of weight load, as shown in Fig. 1b. Secondly, before applying external loads to the escalator, the initial docking port clearances caused by processing and assembly at the positions Y1–Y4 need to be measured by filler gauge after all the bolts at each docking port were tightened. Thirdly, install the dial gauges, the plumb bobs, and the laser range finders according to the marked positions as shown in Fig. 3, and record the current readings.

**Figure 4:**
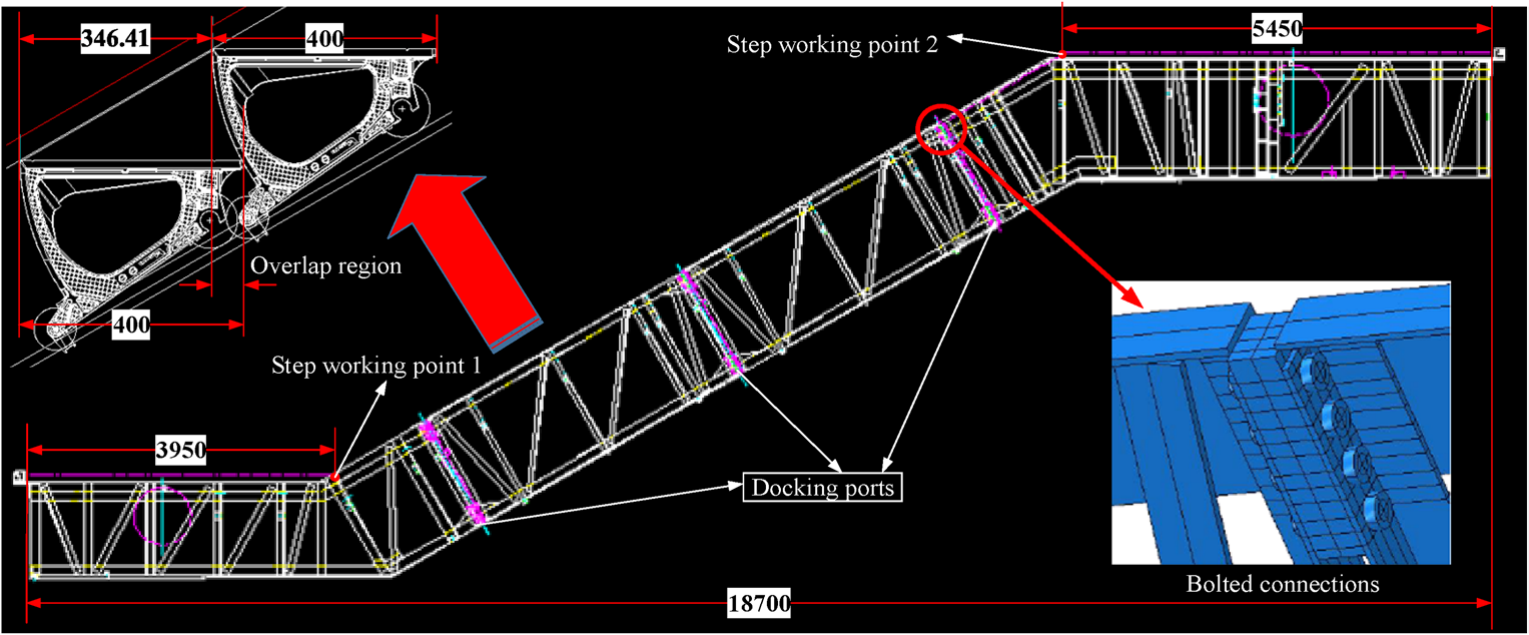
Design of loading scheme.

Then, place weights on the steps and floor plates to apply loads. Figure 4 shows the relevant parameters and lofting graph of the steps. To the left of step working point 1 and to the right of step working point 2, there are horizontal steps and floor plates with a total length of 3950 mm and 5450 mm respectively. While between step working point 1 and step working point 2, the steps tilt upwards with an overlap region between every two steps, which leads to a horizontal projection of the step depth of only 346.41 mm, not 400 mm. Therefore, according to the standard requirements^25^, the load for designing of the escalator support structure comprises the self-weight of the escalator along with an additional load of 5000 N/m^2^. Therefore, when calculating the load, the passenger load need to be applied according to the standard of 5000 N/m^2^ based on the escalator’s horizontal projected area, while the escalator’s self-weight needs to be processed according to the actual situation. So in this work, the horizontal projected area of the escalator *A* is

1 $${\text{A }} = {\text{ H}}_{{\text{s}}} *{\text{ N}}_{{\text{w}}} ,$$

where *H*_*s*_ denotes the horizontal span and *N*_*w*_ represents the nominal width of the escalator. The passenger load of the escalator *P*_*A*_ is

2 $${\text{P}}_{{\text{A}}} = {\text{ S}}_{{\text{r}}} *{\text{A}},$$

where *S*_*r*_ represents the standard load of 5000 N/m^2^. The total weight to be applied *T*_*w*_ is

3 $${\text{T}}_{{\text{w}}} = {\text{P}}_{{\text{A}}} /{\text{g}},$$

where *g* is the gravitational acceleration, and the self-weight of the truss is applied based on it during the simulation. The specific loads to be applied to each part were calculated as shown in Table 4.

**Table 4:** Calculation of the loads to be applied to each part.

Item	Value
Standard requirements (*S*_*r*_)	5000 N/m^2^
Horizontal span (*H*_*s*_)	18,700 mm
Nominal width (*N*_*w*_)	1000 mm
Step depth (*S*_*d*_)	400 mm
Each weight (*E*_*w*_)	20 kg
Gravitational acceleration (*g*)	9.8 m/s^2^
Total weight to be applied (*T*_*w*_)	9540 kg
The length of horizontal steps and floor plates on the left side of the step working point 1 (*L*_*l*_)	3950 mm
Number of weights on the left side of the step working point 1 (*N*_*l*_ = *S*_*r*_ * *L*_*l*_ * *N*_*w*_/g/*E*_*w*_)	101
The length of horizontal steps and floor plates on the right side of the step working point 2 (*L*_*r*_)	5450 mm
Number of weights on the right side of the step working point 2 (*N*_*r*_ = *S*_*r*_ * *L*_*r*_ * *N*_*w*_/g/*E*_*w*_)	139
Horizontal projection length between the step working points (*H*_*p*_ = *H*_*s*_-*L*_*l*_-*L*_*r*_)	9300 mm
Number of weights between the step working points (*N*_*b*_ = *S*_*r*_ * *H*_*p*_ * *N*_*w*_/g/*E*_*w*_)	237
The actual step depth between the step working points (*S*_*ad*_)	346.41 mm
Number of steps between the step working points (*N*_*ss*_ = *H*_*p*_/*S*_*ad*_)	27
Number of weights on each step between the step working points (*N*_*e*_ = *S*_*r*_ * *H*_*p*_ * *N*_*w*_/g/*E*_*w*_/*N*_*ss*_)	9

As can be seen in Table 4, *N*_*l*_ weights need to be evenly placed on the nominal width of the horizontal steps and floor plates on the left side of step working point 1, and *N*_*r*_ weights need to be evenly placed on the nominal width of the horizontal steps and floor plates on the right side of step working point 2. While on the *N*_*ss*_ steps between the step working points, *N*_*b*_ weights need to be evenly placed. If *N*_*e*_ weights are placed on each step, then the total number of weights between the step working points is

4 $$N_{ss} *N_{e} = { 27 }*{ 9 } = { 243},$$

which exceeds 6 weights than *N*_*b*_. To ensure even force, *N*_*e*_ − 1 weights are placed every *N*_*ss*_/(*N*_*ss*_ * N_*e*_ − *N*_*b*_) − 1 step, and *N*_*e*_ weights are placed in the remaining steps. The total weight to be applied to the escalator is

5 $$\left( {N_{l} + N_{r} + \, \left( {N_{ss} - \left( {N_{ss} *N_{e} - N_{b} } \right)} \right)*N_{e} + \, \left( {N_{ss} *N_{e} - N_{b} } \right) \, * \, \left( {N_{e} - 1} \right)} \right) \, *E_{w} = \, 9540\;{\text{kg}},$$

which is consistent with *T*_*w*_. During the loading process, weights need to be placed from the top down to prevent the steps from slipping backward. After placing all the weights on the escalator as required, the escalator should stand still for 30 min to ensure stable deformation.

Finally, according to Table 3, read and record the values of the dial gauges at the positions x1–x4 and y1–y4, and the laser range finders at the positions Y5–Y6, and measure the current positions of each plumb bob, the current docking port clearances at the positions Y1–Y4, and calculate the differences with the initial values to obtain the change values at each measuring position before and after loading.

According to the above procedure, complete the full load test of the experimental escalator with the truss structure depicted in Fig. 2a first. Then, based on the experimental results, the truss structure shown in Fig. 2a was optimized and the new truss structure was obtained as shown in Fig. 2b. To verify that the new truss structure meets the deflection standard, the full load test of the experimental escalator with truss shown in Fig. 2b was performed again, and this time only the docking port clearances near the lower horizontal section and the maximum deflections near the middle of the truss were collected.

### Experiment results and discussion

After loading, the experimental data were collected and processed, and the final experimental results of the escalator with truss structure in Fig. 2a were shown in Table 5, while the final experimental results of the escalator with new truss structure in Fig. 2b were shown in Table 6. To provide the basis for the following parametric study, the experimental results in Table 5 were analyzed from three aspects: the end restraint, the docking port clearance, and the maximum deflection.

**Table 5:** The experimental results of the escalator with truss structure in Fig. 2a.

Position	Horizontal slip	Vertical displacement	Position	Vertical displacement	Docking port clearance
x1	0.65 mm(→)	/	Y1	15.9 mm(↓)	0.24 mm( ↔)
x2	0.50 mm(→)	/	Y2	16.2 mm(↓)	0.22 mm( ↔)
y1	/	1.12 mm(↓)	Y3	17.85 mm(↓)	0.27 mm( ↔)
y2	/	1.00 mm(↓)	Y4	17.26 mm(↓)	0.38 mm( ↔)
x3	0.05 mm(←)	/	Y5	16.0 mm(↓)	/
x4	0.1+ mm(←)	/	Y6	16.0 mm(↓)	/
y3	/	0.65 mm(↓)	Note: Arrow represents the direction (the same as below)		
y4	/	0.50 mm(↓)			
y5	/	1.50 mm(↑)			
y6	/	1.26 mm(↑)

**Table 6:** The experimental results of the escalator with truss structure in Fig. 2b.

Position	Vertical displacement	Docking port clearance	Note
Y1	/	0.1 mm(↔)	On the right side of the lower docking port, filler gauge
Y2	/	0.1 mm(↔)	On the left side of the lower docking port, filler gauge
Y3	11.05 mm(↓)		On the right side near the middle of the truss, plumb bob1
Y4	10.63 mm(↓)		On the left side near the middle of the truss, plumb bob2
Y3	11.4 mm(↓)		On the right side near the middle of the truss, laser range finder 1
Y4	11.3 mm(↓)		On the left side near the middle of the truss, laser range finder 2

#### End restraint

Usually, the end-supported beams are considered fixed to the civil structure, and will not produce slip when subjected to external loads. Therefore, in previous simulation studies on truss structure, constraints such as fixed-end support are mostly used, leading to conservatively cautious simulation outcomes. It wasn't until the experimental results were available that the unsatisfactory nature of these outcomes became evident, ultimately causing significant economic losses. However, the choice of end restraint type exerts a considerable influence on the total deflection, and there has been little research on determining the type of end restraint. Based on this, experimental research was carried out to fill this gap in this work.

Before the experiments, we have conducted a simulation comparison using the truss structure model in Fig. 2b to examine the effect of varying support conditions. The outcomes of this comparison are presented in Table 7. It can be observed that the type of end constraint does have a significant impact on the deflection. The simulation result under the fixed-end support condition is smallest among all the simulation results, while the simulation result under the horizontal slip and rotation support condition is the closest to the experimental result, with an error of 10.79%. Therefore, to corroborate the hypothesis of horizontal slip and rotation support condition, experimental validation is very necessary.

**Table 7:** Comparison of simulation results under different support conditions and the experimental result with truss structure in Fig. 2b.

Maximum deflection/mm					
Method	Simulation				Experiment
End restraint type	Fixed-end	Rotation without horizontal slip	Horizontal slip without rotation	Horizontal slip and rotation	/
Result	9.33	9.54	10.00	10.17	11.40 (average)
Absolute error	− 2.07	− 1.86	− 1.40	− 1.23	0
Relative error	18.14% (↓)	16.36% (↓)	12.31% (↓)	10.79% (↓)	0

In Table 5, the significant horizontal slips at the positions x1–x4 and vertical displacements at the positions y1–y6 can be observed after loading. It can be seen that the horizontal slip and vertical displacement measured on the right side are generally larger than those on the left side, which is mainly caused by the unbalanced placement of weights. At the same time, the opposite directions of horizontal slip at the right and the left ends indicates that under the action of external load, the end support beams slide to the middle of the escalator, while the part between the end support beams deforms downward. And the opposite directions of vertical displacement at the positions y1, y2 and y6 indicates that under the action of external load, the end support beam in both the lower and upper horizontal sections experiences rotation. The above results indicate that the constraints of fixed-end support used in the previous simulation studies on truss structure are inconsistent with the actual measurement results, which need to be corrected. On this basis, the mode of setting the end restraint as horizontal slip and rotation is considered in the subsequent parametric study.

#### Docking port clearance

As mentioned before, the truss comprises a lower horizontal section, several straight sections, and an upper horizontal section. To ensure the overall strength of the truss, high strength bolt connections between the sections are required. In this work, four sets of high strength bolts were installed at each docking port of the experimental escalator, each consisting of four M24 bolts with 10.9 s strength grade arranged side by side as shown in Fig. 4. And the pretension force of each bolt in Fig. 2a was applied through the torque wrench with 500 Nm torque, which is equivalent to 94 kN.

As can be seen in Table 5, after loading, the docking port clearances measured at the positions Y1–Y4 increased by 0.24 mm, 0.22 mm, 0.27 mm, and 0.38 mm respectively, which means that the docking ports were pulled apart under this kind of load condition, and also indicates that the pretension force applied to each bolt was insufficient. So for the truss structure in Fig. 2b, the pretension force of each bolt was applied through the torque wrench with 700 Nm torque, which increased to 133 kN, and the docking port clearances measured at the positions Y1–Y2 reduced to 0.1 mm, and the overall structural strength of the truss was enhanced. But the question of how much the pretension force needs to be applied to each bolt is still worth investigating because the greater the pretension force, the more difficult it is to operate in a narrow space. In this work, numerical simulation was performed to solve this problem.

#### Maximum deflection

According to the standard requirements, the maximum deflection measured or calculated should not be greater than 1/1500 of the horizontal span between the supporting points. In this work, the horizontal span between the supporting points is *H*_*s*_ = 18,700 mm, so the maximum deflection standard is

6 $$[\upgamma ] = { 1}/{15}00 \, *H_{s} = { 12}.{47}\;\;{\text{mm}}$$

As can be seen in Table 5, the maximum vertical displacement measured at positions Y1–Y6 after loading was 17.85 mm, which appeared at position Y3, namely the right side of the intermediate docking port. It goes well beyond the maximum deflection standard [γ], meaning that the truss structure in Fig. 2a is not qualified and needs to be strengthened, which may be achieved through the measures such as removing the intermediate docking port, increasing the pretension force of each bolt, welding the middle soffit plate to make it into a whole, thickening or extending the chord reinforcement plate, etc. Comparing the measured values at positions Y4 and Y5, it can be found that the result measured by the laser range finder is smaller than that measured by the plumb bob. The main reason may be that the laser range finder pasted on the lower chord has a small angle deflection with the deformation of the truss structure during the loading process.

While for the truss structure in Fig. 2b, the maximum vertical displacement measured at the positions Y1–Y4 after loading was 11.4 mm, less than the maximum deflection standard [γ], meaning that the new truss structure in Fig. 2b is qualified. Therefore, the truss structure in Fig. 2b will be taken as the prototype, and the experimental results in Table 6 as the benchmark to carry out the subsequent parametric study.

## Simulation calculation

### Simulation analysis for the truss structure

**Figure 5:**
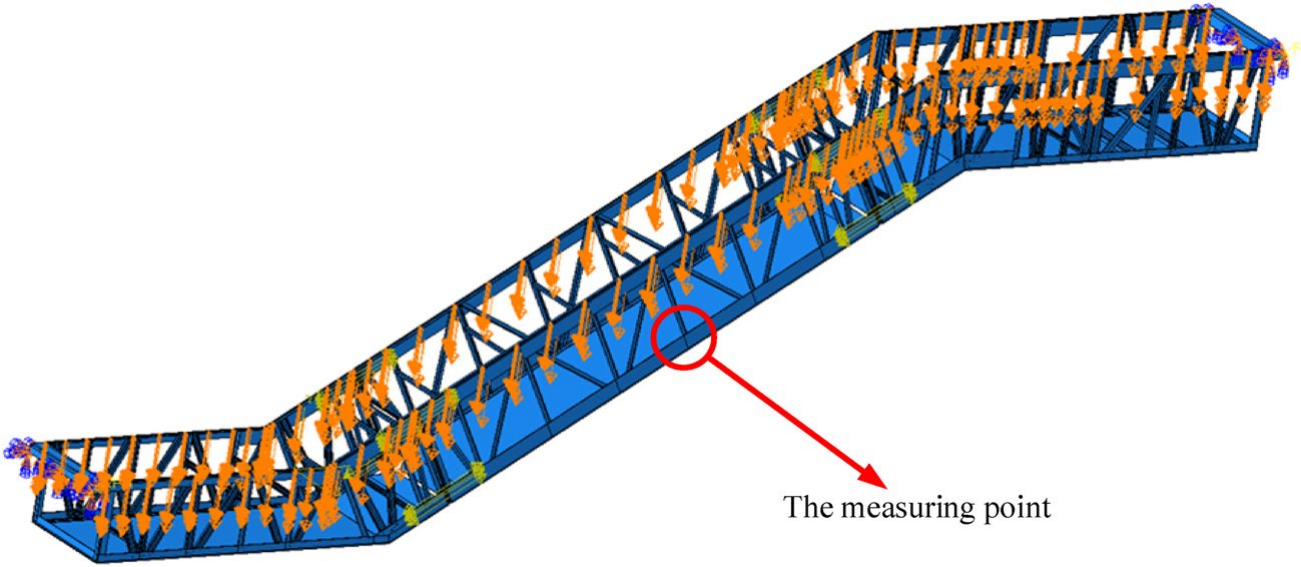
The three-dimensional simulation model of the truss.

The reliability of the numerical simulation results depends on many factors, such as the degree of simplification of the model, the setting of boundary conditions, the way of loading, and so on. To improve the accuracy of simulation calculations, the finite element simulation software ABAQUS Version 2020 was used to carry out static analysis based on the previous experimental results. According to the truss structure in Fig. 2b, the three-dimensional simulation model of the truss was established with an 8-node linear hexahedron element (C3D8R), as illustrated in Fig. 5. Through iterative optimization, the simulation model can achieve the maximum reduction of the truss of the experimental escalator by aligning simulation results with experimental data. At the same time, the impact of diverse factors on the deflection of the truss was analyzed.

**Figure 6:**
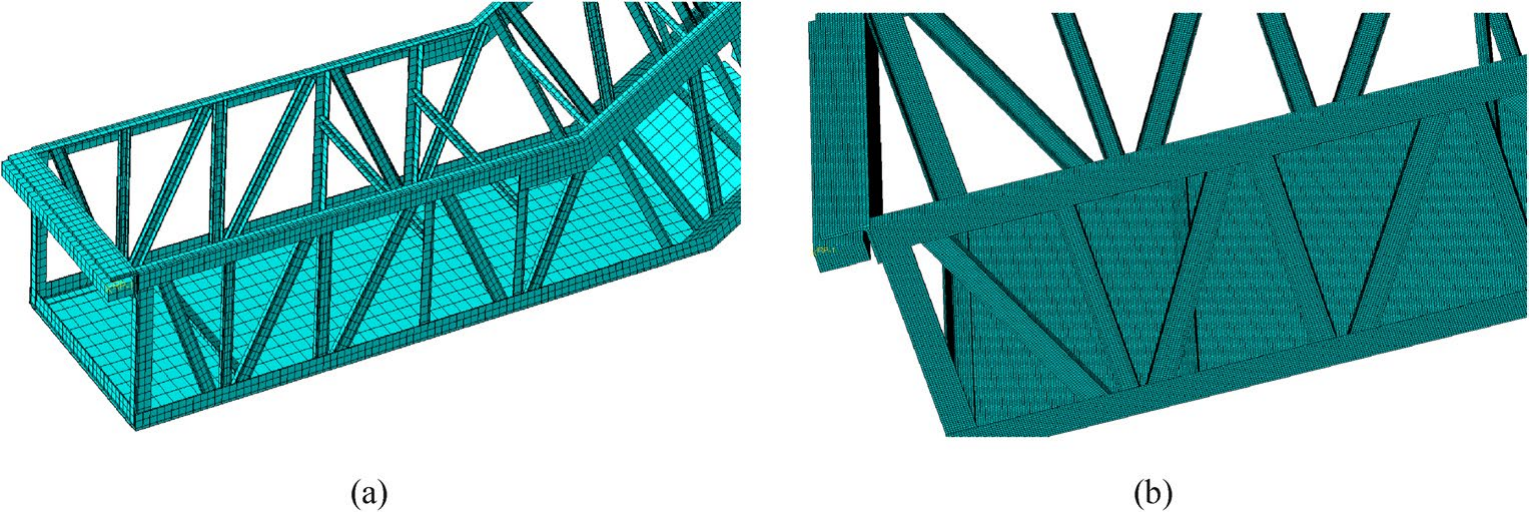
Comparison of mesh division in the lower horizontal section: (**a**) size 60 mm; (**b**) size 10 mm.

After creating the simulation model, the material parameter of each truss member was set to refer the introduction in "Detailed information about the experimental object" section. Then, the finite element model needs to be meshed. To improve computing efficiency, the mesh size used to analyze the escalator truss structure is usually set above 60 mm. In this work, the smaller mesh sizes were employed to examine the impact of mesh size on deflection and identify a stable range. Figure 6 shows the mesh division in the lower horizontal section with different sizes. The results of these analysis were summarized in Table 8. It can be clearly observed that the smaller the finite element mesh size is, the higher the calculation accuracy will be. With the mesh size decreasing from 60 to 10 mm, the maximum deflection of the truss increases from 8.48 to 10.17 mm, close to the measured value. Meanwhile, when the mesh size is small to a certain extent, the calculation results of the model tend to be stable. However, the calculation time with the mesh size of 10 mm was more than twice that with the mesh size of 60 mm. The calculation time is unacceptable for using a mesh size smaller than 10 mm to simulate. Therefore, the mesh size of all models in this work was divided into 10 mm to enhance the computational efficiency while ensuring sufficient computational accuracy.

**Table 8:** The influence of mesh size on the maximum deflection.

Mesh size/mm	60	40	30	20	10
Deflection/mm	8.48	9.05	9.60	10.37	10.17
Relative error	16.57% (↓)	11.05% (↓)	5.61% (↓)	1.92% (↑)	0

According to the experimental results in "End restraint" section, the end restraint was set as horizontal slip and rotation, that is, applying Y- and Z-direction translational constraints, as well as X- and Y-direction rotational constraints on the upper and lower end support beams, respectively, while retaining only X-direction translational freedom and Z-direction rotation freedom. The initial pretension force of each bolt was set as 133 kN.

According to the standard requirements, the maximum deflection is determined by calculating or measuring it under the condition of applying a load of 5000 N/m^2^ on the escalator’s horizontal projected area. Since the truss is a frame structure, the load can be applied to the joints of the upper chord, which needs to be converted into concentrated forces when applied to the finite element model. The specific load calculation was listed in Table 9. Then, apply the concentrated forces with the direction straight down according to the calculated values and run the simulation. After the calculation, the deformation results at the measuring point in Fig. 5 was extracted as the deflection value for comparison with the experimental result at position Y3.

**Table 9:** The specific load calculation of each joint.

Item	Value
Standard requirements (*S*_*r*_)	5000 N/m^2^
Horizontal span (*H*_*m*_)	18,601 mm
Nominal width (*N*_*w*_)	1000 mm
The length of the upper chord of the lower horizontal section (*L*_*ul*_)	3691 mm
The total load on the lower horizontal section (*T*_*tl*_ = *S*_r_*L_ul_*N_*w*_)	18,455 N
Number of joints on the upper chord of the lower horizontal section (*N*_*jl*_)	12
The concentrated force of joints on the upper chord of the lower horizontal section (*F*_*jl*_ = *T*_*tl*_/*N*_*jl*_)	1537.92 N
The length of the upper chord of the upper horizontal section (*L*_*uu*_)	5609 mm
The total load on the upper horizontal section (*T*_*tu*_ = *S*_r_*L_uu_*N_*w*_)	28,045 N
Number of joints on the upper chord of the upper horizontal section (*N*_*ju*_)	18
The concentrated force of joints on the upper chord of the upper horizontal section (*F*_*ju*_ = *T*_*tu*_/*N*_*ju*_)	1558.06 N
The horizontal projection length of the upper chord of the inclined section (*L*_*ui*_ = *H*_*m*_-*L*_*ul*_-*L*_*uu*_)	9301 mm
The total load on the inclined section (*T*_*ti*_ = *S*_r_*L_ui_*N_*w*_)	46505 N
Number of joints on the upper chord of the inclined section (*N*_*ji*_)	32
The concentrated force of joints on the upper chord of the inclined section (*F*_*ji*_ = *T*_*ti*_/*N*_*ji*_)	1453.28 N

Table 10 shows the comparison between the initial simulation and the experimental result. It can be seen that simulation result (A) is very close to the experimental result, indicating that the created three-dimensional simulation model is valid. The relative error is − 10.79%, indicating that the stiffness of the model increases with the discretization, and meanwhile, some influencing factors may not have been taken into account during the modeling process. Based on this, the factors affecting the deflection and their degree of influence were explored in the next section.

**Table 10:** The comparison between the initial simulation and the experimental result.

Item	Deflection value	Relative error
The experimental result	11.4 mm	/
The simulation result (A)	10.17 mm	− 10.79%

**Figure 7:**
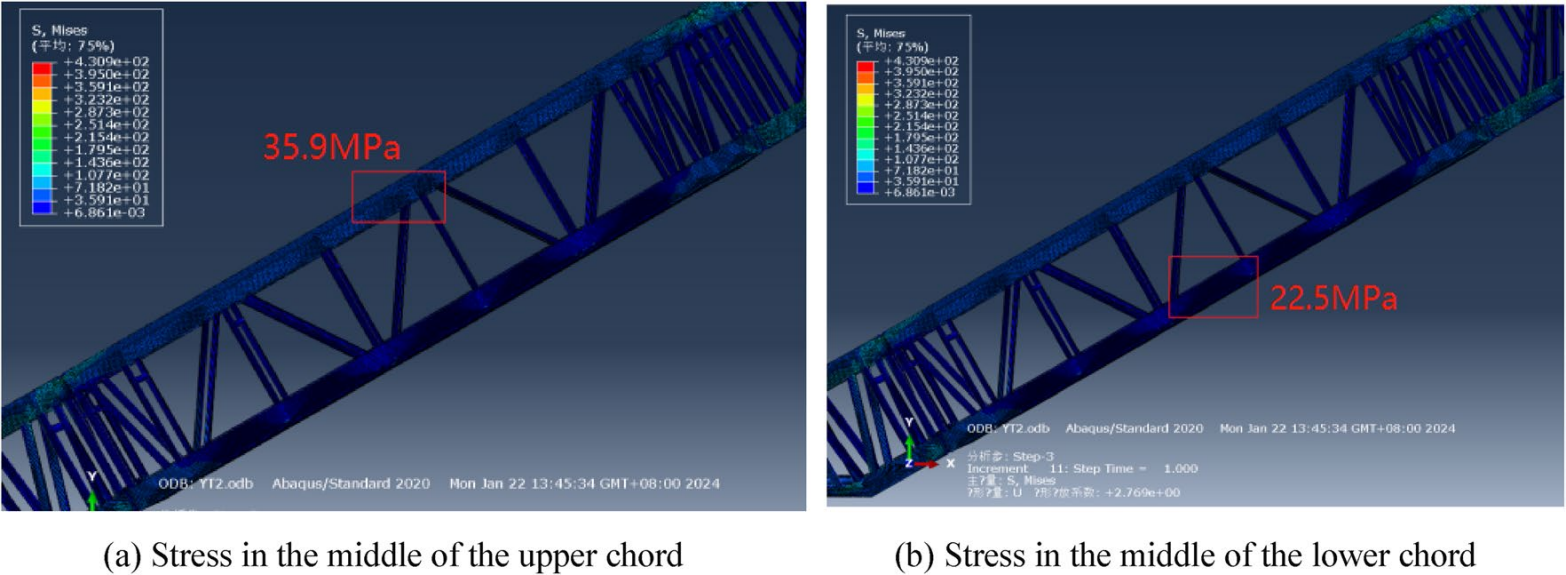
Stress distribution in the intermediate inclined section.

The stress distribution is a crucial aspect to consider for escalator safety. In past practical tests, the strength of the escalator truss was typically evaluated using the strain gauge method, which is widely utilized in engineering testing. Prior to loading, strain gauges were affixed to the middle of the upper and lower chords (after appropriate surface preparation). Following loading, the stress values at these locations were recorded. Nevertheless, the current owner does not deem the truss strength test as a mandatory requirement during the acceptance of the escalator. The primary rationale behind this is that based on the computational experiences (shown in Fig. 7), and disregarding the stress concentration issues associated with simplified models, the typical stress in the truss remains below 40 MPa, resulting in a significantly large safety factor (≥ 5). For these reasons, the strength indicators were not incorporated into the scope of this study.

**Figure 8:**
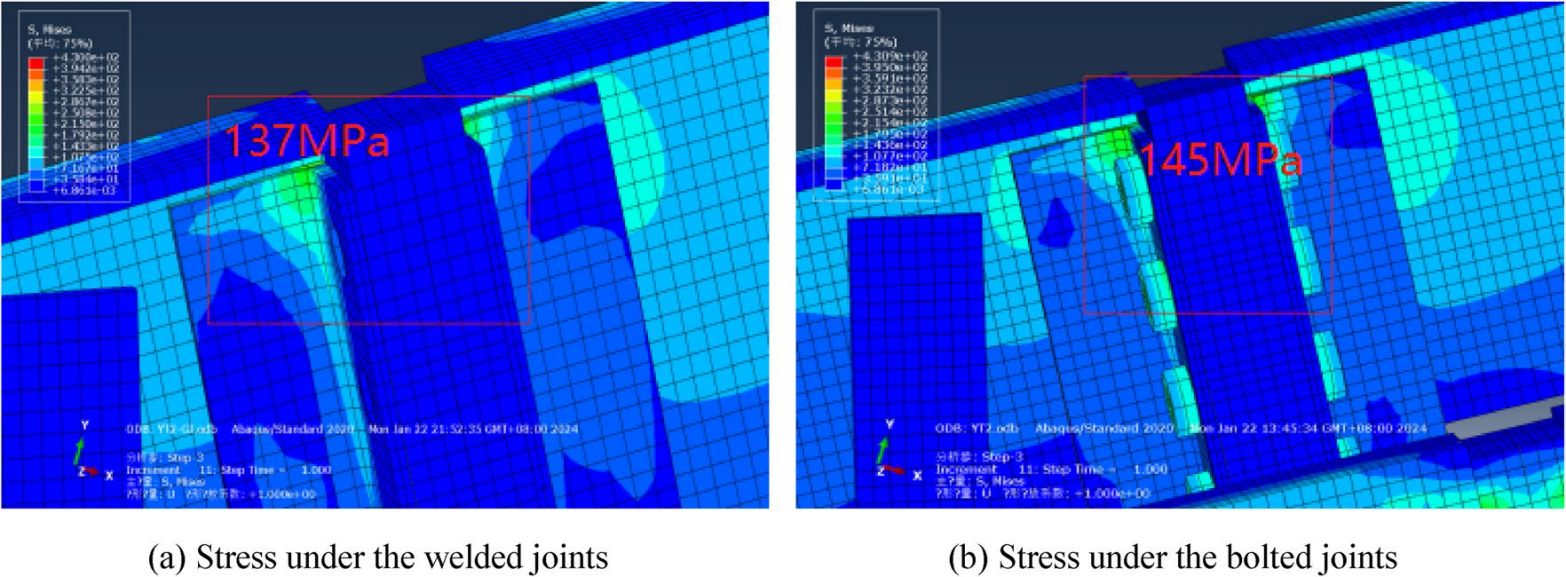
Stress distribution at the docking ports.

In order to study the degree of influence of bolted joints on deflection, the solid models with welded and bolted joints were established and the stress distribution at the docking ports is depicted in Fig. 8. It is evident that the stress distribution at the docking ports is highly similar under the two connection methods. Specifically, the stress under the welded joints is slightly lower than that under the bolted joints, about 137 MPa. This phenomenon is mainly due to the fact that the welded joints improve the tensile strength of the overall structure. Under the bolted joints, the tensile stress borne by the bolt is about 145 MPa, far below its yield strength of 900 MPa, so it is also in a safe state. The computed deflection value are presented in Table 11. Compared with the welded joints solid model, the deflection of the bolted joints solid model has increased, however, the influence of the bolted joints on deflection is relatively small. To guarantee the precision of the calculation results, the subsequent simulation model will uniformly adopt the bolt connection method to maintain consistency with the physical connection method.

**Table 11:** The comparison under different connection forms.

Item	Deflection value/mm	Relative error
Under the welded joints	10.07	/
Under the bolted joints	10.17	0.97%

### Parametric study of escalator truss structure

To investigate the main sources of the above error, a comparative analysis was conducted between the three-dimensional simulation model and the experimental truss structure of the heavy-duty escalator. It can be found that there were differences in the parameters such as profile section chamfer setting, and lower deviation setting of profile size, etc. At the same time, the simulation calculation did not consider the influence of the dead weight of the escalator, which may also lead to the difference. The following discussions were carried out from these aspects. At last, the question of how much the pretension force needs to be applied to each bolt was explored.

#### Influence of the profile section chamfer of the truss member

**Figure 9:**
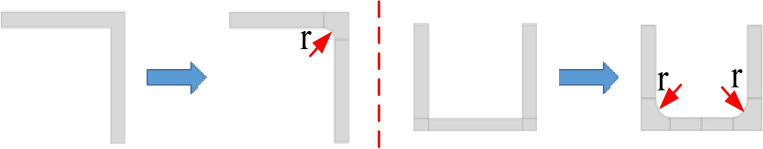
The profile section chamfer of the truss member.

As mentioned before, the truss members are mainly made of the profiles such as angle steel, channel steel, and plate. In the actual stress process, these profiles in the truss mainly bear the axial force, so the cross-sectional size of these profiles should be as close as possible to the actual situation during the modeling process. It is well known that angle steel and channel steel have internal chamfers and edge chamfers, and the flange of the channel steel has a slope of 1/10. However, these features have been simplified in the previous modeling process. Considering that the edge chamfer and flange slope are not conducive to grid division in the model, and the model is difficult to converge, the edge chamfer and flange slope are neglected generally in the modeling process. In this work, only the effect of the internal chamfers on the deflection was investigated. Referring to the standard GB/T 706^26^, the model of the truss members was reconstructed as demonstrated in Fig. 9. The internal chamfer radius of each truss member was shown in Table 12.

**Table 12:** The section parameters of truss members.

Type	Internal chamfer radius/mm	Lower deviation of profile size		
		Thickness/mm	Width/mm	Height/mm
∠200 * 200 * 24	18	− 1.0	− 2.5	/
∠125 * 80 * 8, ∠125 * 80 * 10	11	− 0.7	− 2.0	/
∠80 * 80 * 8, ∠80 * 80 * 10	9	− 0.6	− 1.2	/
∠63 * 63 * 6	7	− 0.6	− 1.2	/
⊏100 * 48 * 5.3	8.5	− 0.5	− 2.0	− 2.0
⊏80 * 43 * 5	8	− 0.4	− 1.5	− 1.5
⊏63 × 40 × 4.8	7.5	− 0.4	− 1.5	− 1.5
The soffit plate	/	− 0.55	/	/

Keep the other conditions unchanged and perform this simulation. The calculation result was listed in Table 13. It was found that the deflection value decreased from 10.17 to 9.85 mm after taking the internal chamfers of each truss member into account. This result was caused by the increase in cross-sectional area, evident in Fig. 9, which subsequently enhances the bending stiffness of the truss. At last, the relative error between the simulation result (B) and the experimental result returned to − 13.60%.

**Table 13:** The influence of the profile internal chamfer of truss member.

Item	Deflection Value	Relative error
The experimental result	11.4 mm	/
The simulation result (B)	9.85 mm	− 13.60%

#### Influence of the lower deviation of profile size of the truss member

Under the premise of ensuring standard requirements, material manufacturers often produce profiles according to the lower deviation of the standard size to reduce the production cost of raw materials. While the trusses made of the profiles with these kinds of lower deviation dimensions often need to be reinforced to meet the deflection standard requirement. The previously established model was created based on the standard size without considering the effect of the lower deviation. To this end, a new model consisting of the profiles with lower deviations was re-established with reference to the standard GB/T 706^26^. The lower deviation parameters of each truss member were also shown in Table 12.

Keep the other conditions unchanged and perform this simulation. The calculation result was listed in Table 14. It can be observed that the deflection value increased from 9.85 to 11.13 mm after considering the lower deviations, which means the effect of the lower deviations on the deflection value is significant. The reason was the same as the situation of the decrease in the width of the soffit plate in "Influence of the profile section chamfer of the truss member" section. At last, the relative error between the simulation result (C) and the experimental outcome reduced to a mere − 2.37% with the difference almost negligible. Simultaneously, a comparison was made between the truss mass with and without considering the lower deviations of the profiles. It was found that the mass was reduced by approximately 8.75%, about 440 kg, when considering the lower deviations. On the premise of ensuring the standard requirements, producing the profiles with lower deviations can indeed save a lot of material cost.

**Table 14:** The influence of the lower deviation of profile size of truss member.

Item	Deflection value	Relative error	Truss mass	Relative error
The experimental result	11.4 mm	/	/	/
The simulation result (B)	9.85 mm	− 13.6%	5.03 t	/
The simulation result (C)	11.13 mm	− 2.37%	4.59 t	− 8.75%

#### Influence of the dead weight of the escalator

As mentioned before, the relative measuring method was adopted to measure the deflection value of the experimental escalator in this work because the dead weight of the escalator itself caused the truss to deform before the external load was applied, while in the simulation, the deflection value caused by the applied external load was directly calculated, without considering the influence of the dead weight of the escalator on the deflection calculation value. To explore its degree of influence, the deflection values under two kinds of load conditions were calculated: (a) with a load of the overall dead weight of 15 t, (b) with the load of the overall dead weight of 15 t, and the external load of 5000 N/m^2^. All the loads were applied on the upper chords of the truss. Keep the other conditions unchanged and perform these simulations. The deflection value was the difference between the simulation result under condition b) and the simulation result under condition a).

The comparison results with and without considering the dead weight of the escalator were shown in Table 15. It can be observed that the deflection value considering the dead weight of the escalator increased by 8.63%, which means the dead weight of the escalator has a significant effect on the deflection value. The reason may be that the end support beams not only slip horizontally but rotate due to the action of the dead weight and external load, resulting in a nonlinear change of the deformation. Compared with the experimental result, the simulation result (B) without the lower deviations but considering the dead weight differed by − 6.14%, while the simulation result (C) with the lower deviations and considering the dead weight differed by 6.05%. On the whole, the influence of the dead weight of the escalator cannot be neglected during the simulation.

**Table 15:** The influence of the dead weight of the escalator.

Item	Deflection value without dead weight (mm)	Deflection value with dead weight (mm)	Relative value (%)
The simulation result (B) without the lower deviations	9.85	10.70	8.63
The simulation result (C) with the lower deviations	11.13	12.09	8.63

#### Influence of the pretension force applied to each bolt at the docking ports

As mentioned before, the truss is connected by the high strength bolts between each section. The amount of the pretension force applied to each bolt will affect the overall strength of the truss. Due to the small operating space, it is difficult to apply a large pretension force. Therefore, the appropriate pretension force needs to be determined.

**Figure 10:**
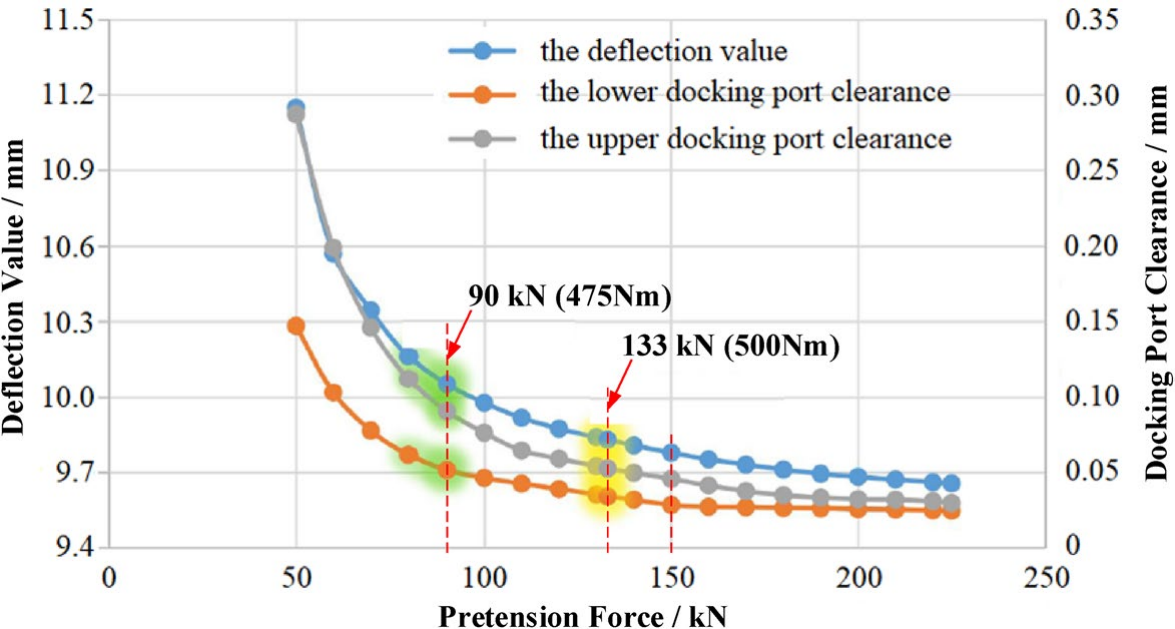
The effect of the pretension force on the structural property of truss.

According to the standard JGJ 82^27^, the maximum pretension force for the M24 bolt with a 10.9 s strength grade is 225 kN. To investigate the influence of the pretension force applied to each bolt on the structural performance of the truss, the calculation point was set every 10 kN within the range of 50–225 kN. Keep the other conditions unchanged and perform these simulations. The influence curves of the pretension force on the deflection value and the docking port clearances were shown in Fig. 10. It can be seen that with the increase of the pretension force, the deflection value and the docking port clearances decrease gradually, and the curves tend to be smooth when the pretension force was 150 kN, which is equivalent to the load applied through the torque wrench with 792 Nm torque, based on the bolt pretension force formula

7 $$M_{t} = K*P_{0} *d$$

where *M*_*t*_ is the pretension torque, Nm; *K* is the tightening force coefficient; *P*_*0*_ is the bolt pretension force, kN; *d* is the bolt diameter, mm. For the galvanized non-lubricated contact surfaces, *K* was set to 0.22, and the diameter *d* for M24 bolts is 24 mm. Therefore, for the truss similar to that of the experimental escalator, a pretension force of 150 kN applied to each bolt was suggested.

## Conclusions

The experimental studies on the structural performance of heavy-duty escalators under full load conditions were performed in this work. During the experiments, the horizontal slips, the vertical displacements as well as the docking port clearances at different positions were measured with different methods. Based on the experimental results, the end restraint, the stress at the docking ports, and the maximum deflection were analyzed. The three-dimensional finite element models considering the horizontal slip and rotate end restraint were established to investigate the influences of various factors on the deflection of the escalator truss, such as the internal chamfer, the lower deviation of the truss member, the dead weight of escalator, and the pretension force of each bolt. By synthesizing the experimental and simulation results, the subsequent conclusions can be deduced.

1. Under the action of external load, the end support beams slide to the middle of the escalator, while the part between the end support beams deforms downward. At the same time, the end support beams also undergo rotation. The end restraints are suggested to be set as horizontal slip and rotation in the simulation study on truss structure.
2. Taking the effect of the lower deviations into consideration will result in a decrease in the inertia moment of the whole section of the truss, which leads to a decrease in the bending stiffness of the truss, and thus the deflection value increases. However, the influence of the profile internal chamfer of truss members is opposite.
3. The dead weight of the escalator has a significant effect on the deflection value, which cannot be neglected during the simulation. The reason may be that the end support beams not only slip horizontally but rotate due to the action of the dead weight and external load, resulting in a nonlinear change of the deformation.
4. The docking ports of the truss were pulled apart during the experiments, and the pretension force applied to each bolt was suggested to be set as 150 kN. With the increase of the pretension force, the deflection value and the docking port clearances decrease gradually.

The above findings can provide the basis for the efficient design, correct simulation, low cost production and rapid installation of escalator truss. The upcoming work will primarily concentrate on optimizing the design of the escalator's truss structure, such as the design of free segments, the arrangement of inclined bars, the member arrangement of inflection points, the design of member interval, the design of cross-section height, the analysis of truss width, and the design of reinforcement or weakening schemes for trusses.

## Data Availability

The data will be available upon request. The corresponding author (SXL) should be contacted for this purpose.
